# Glycan binding patterns of human rotavirus P[10] VP8* protein

**DOI:** 10.1186/s12985-018-1065-9

**Published:** 2018-10-19

**Authors:** Li-li Pang, Meng-xuan Wang, Xiao-man Sun, Yue Yuan, Yu Qing, Yan Xin, Jia-yan Zhang, Dan-di Li, Zhao-jun Duan

**Affiliations:** 1 National Institute for Viral Disease Control and Prevention, Chinese Center for Disease Control and Prevention, Beijing, 102206 China; 2 Key Laboratory of Medical Virology and Viral Diseases, Ministry of Health of the People’s Republic of China, Beijing, 102206 China; 3 Department of Food and Chemical Engineering, Lushan College of Guangxi University of Science and Technology, Liuzhou, 545616 Guangxi China; 4 Basic Medical College, Inner Mongolia Medical University, Hohhot, 010000 China

**Keywords:** Rotavirus, P [10] genotype, VP8*, Mucin core, Oligosaccharide binding assay

## Abstract

**Background:**

Rotaviruses (RVs) are a major cause of acute children gastroenteritis. The rotavirus P [10] belongs to P[I] genogroup of group A rotaviruses that mainly infect animals, while the rotavirus P [10] was mainly identified from human infection. The rotavirus P [10] is an unusual genotype and the recognition pattern of cellular receptors remains unclear.

**Methods:**

We expressed and purified the RV P [10] VP8* protein and investigated the saliva and oligosaccharide binding profiles of the protein. A homology model of the P [10] VP8* core protein was built and the superimposition structural analysis of P [10] VP8* protein on P [19] VP8* in complex with mucin core 2 was performed to explore the possible docking structural basis of P [10] VP8* and mucin cores.

**Results:**

Our data showed that rotavirus P [10] VP8* protein bound to all ABO secretor and non-secretor saliva. The rotavirus P [10] could bind strongly to mucin core 2 and weakly to mucin core 4. The homology modeling indicated that RV P [10] VP8* binds to mucin core 2 using a potential glycan binding site that is the same to P [19] VP8* belonging to P[II] genogroup.

**Conclusion:**

Our results suggested an interaction of rotavirus P [10] VP8* protein with mucin core 2 and mucin core 4. These findings offer potential for elucidating the mechanism of RV A host specificity, evolution and epidemiology.

## Background

Human rotaviruses (RVs) are the major etiological agent of acute gastroenteritis in infants and young children worldwide. Rotavirus can cause severe gastroenteritis, and rotavirus-related diarrhea leads to about 450,000 deaths globally each year [1, 2]. Rotavirus, belonging to the *Reoviridae* family, possesses 11 segments of double-stranded RNA encoding 12 proteins, including six structural proteins (VP1, VP2, VP3, VP4, VP6, and VP7) and six nonstructural proteins (NSP1 to NSP6). Based on the sequence variability of the outer capsid glycoprotein VP7 and protease-sensitive spike protein VP4, group A rotavirus, the most common species, is classified into various G and P types respectively. The current evidence indicates that there exists at least 27 G and 37 P genotypes [3, 4]. G1-G4, G9, G12, P [4], P [6], and P [8] are considered as the common human rotavirus genotypes [5].

The rotavirus P [10] is an unusual genotype, which was first identified in a human G8 RVA strain [6, 7]. Since the first report in 1984, rotavirus P [10] strains have been detected occasionally with different G genotypes from different countries. The human P [10] rotavirus prototype strain 69 M as well as B37 were G8P [10]. There were G9P [10] strains found in Ghana [8]. P [10] was also found in combination with G3 human genotype with severe infantile diarrhea [9, 10]. Group A rotaviruses can infect both humans and animals. The P [10] rotavirus is predominantly found in humans. In animals, P [10] strain was identified from swine in Denmark with unknown VP7 genotype, and a strain of G3P [10] was identified from a bat in China [11, 12].

The first step of rotavirus infection involves the recognition of specific cell surface glycans in the initial cell attachment step. Recent studies indicate that RVs recognize histo-blood group antigens (HBGAs) as potential receptors [13–15]. The attachment of RV to cell surface carbohydrates is mediated by the VP8* domain of the spike protein VP4. RV with different genotypes recognizes various carbohydrate ligands. Significant advances in understanding ligand-associated RVs have been made [16–19]. P [10] was implied to possess the similar glycan binding specificity to P [19] VP8*. However, the specific glycan binding pattern and the interaction mechanisms of human rotavirus P [10] with the glycans remains limited. In this study, we explored the characterization of glycan binding specificity of human rotavirus P [10] by various assays.

## Methods

### Expression and purification of VP8* protein in *Escherichia coli*

The VP8* gene segment from a human P [10] strain, RVA/Human-wt/IND/mcs60/2011/G3P [10] (GenBank accession number JQ358765.1), was synthesized by Genewiz Company (Suzhou, China). The full VP8* gene (encoding amino acids 1 to 232) was cloned with an N-terminal glutathione S-transferase (GST) tag into a pGEX4T-1 expression vector. The vector was transformed into *Esherichia coli* strain BL21 (DE3) and then the expression of protein was induced with isopropyl-β-D-thiogalactopyranoside (IPTG) at a final concentration of 0.4 mM at 22 °C for 16 h. The recombinant protein was purified using Glutathione Sepharose 4 Fast Flow (GE Healthcare Life Sciences) as reported previously [20, 21]. Briefly, the supernatant of the bacterial lysate was bound to glutathione-Sepharose for 2 h at room temperature after ultrasonication for 30 min. The beads were washed five times with phosphate-buffered saline (PBS). The GST fusion protein of interest was eluted with elution buffer (10 mM reduced glutathione, 50 mM Tris-HCl, pH 8.0). Samples of eluted product were subjected to sodium dodecyl sulfate-polyacrylamide gel electrophoresis (SDS-PAGE) to check the proteins. GST-VP8* protein was concentrated to ~ 6 mg/ml using the 10-kDa concentration tube (Millipore), centrifuged at a speed of 2,000×*g*. The VP8* protein (amino acids 1 to 230) of human P [19] Mc345 (GenBank: D38054) was conserved in our lab [20].

### Saliva binding assay

A panel of saliva samples with typed A, B, and O and secretor status was kept in our laboratory. HBGA phenotypes of these saliva samples have been determined by enzyme immunoassays as previously described [22]. The saliva samples were boiled for 10 min and centrifuged at a speed of 1000 rpm for 5 min and then diluted by 1:1000 with PBS. The 96-well microtiter plate was coated with saliva samples at 4 °C overnight and then blocked with 5% nonfat milk. The purified GST-VP8* fusion protein of human P [10] (20 μg/ml) was added at a volume of 100 μl per well. GST protein was included as a negative control. Next, mouse GST antibody (1:1000; Abcam) was added, and the bound antibody was detected using the horseradish peroxidase (HRP)-conjugated goat anti-mouse antibody (1:1,500; Abgent). The plates were incubated at 37 °C for 1 h and washed five times with 0.05% PBS-Tween 20 buffer at each step. The reaction was developed using a 3,3′,5,5′-tetramethylbenzidine (TMB) kit (Invitrogen), and the absorbance at 450 nm was determined within 5 min using a microplate reader.

### Oligosaccharide binding assay

The purified GST-VP8* fusion proteins of human P [10], P [19], P [14] and GST protein were diluted with PBS at 200 μg/ml separately. The 96-well microtiter plate was coated with the diluted GST-VP8* at a volume of 100 μl per well at 4 °C overnight and then blocked with 5% nonfat milk, as reported previously [17, 20]. Synthetic-oligosaccharide-polyacrylamide (PAA)-biotin conjugates (Lewis a [Le^a^], Le^b^, Le^c^, Le^x^, Le^y^, A, B, H1, H2, H3, α_a_β_1,4_, mucin core 2, mucin core 4, mucin core 6, Neu5Ac, Neu5Gc) (GlycoTech) were added at 0.2 μg per well at 4 °C overnight. Then, HRP-conjugated streptavidin (Abcam) was added at 0.1 μg per well at 37 °C for 1 h. At each step, the plates were washed five times with 0.5% PBS-Tween 20 buffer. The plate was added with 100 μl TMB Substrate Solution and incubation in the dark for 15 min. Finally, 100 μl Stop Solution were added to each well, and the absorbance at 450 nm was determined within 5 min.

### Homology modeling of P [10] VP8*core and superimposition structural analysis

A homology model of the P [10] VP8* core protein was built based on the X-raycrystal structure of the rhesus rotavirus VP4 sialic acid binding domain in complex with 2-O-methyl-alpha-D-N-acetyl neuraminic acid (SMT ID: 1kqr.1) as the template by SWISS-MODEL automated protein structure homology modeling server (http://swissmodel.expasy.org/). Superimposition structural analysis of P [10] VP8* protein on P [19] VP8* in complex with core 2 (PDB identifier [ID] 5VKI) was performed using the PyMOL software package (https://pymol.org/2/).

### Ethics statement

The study was conducted with the approval of the Institutional Review Boards of National Institute for Viral Disease Control and Prevention, Center for Disease Control and Prevention of China (No. IVDC2015–011). Informed consent was obtained from the members who provided samples. All experiments were performed in accordance with relevant guidelines and regulations of China.

## Results

### Expression and purification of P [10] VP8* protein

**Fig. 1:**
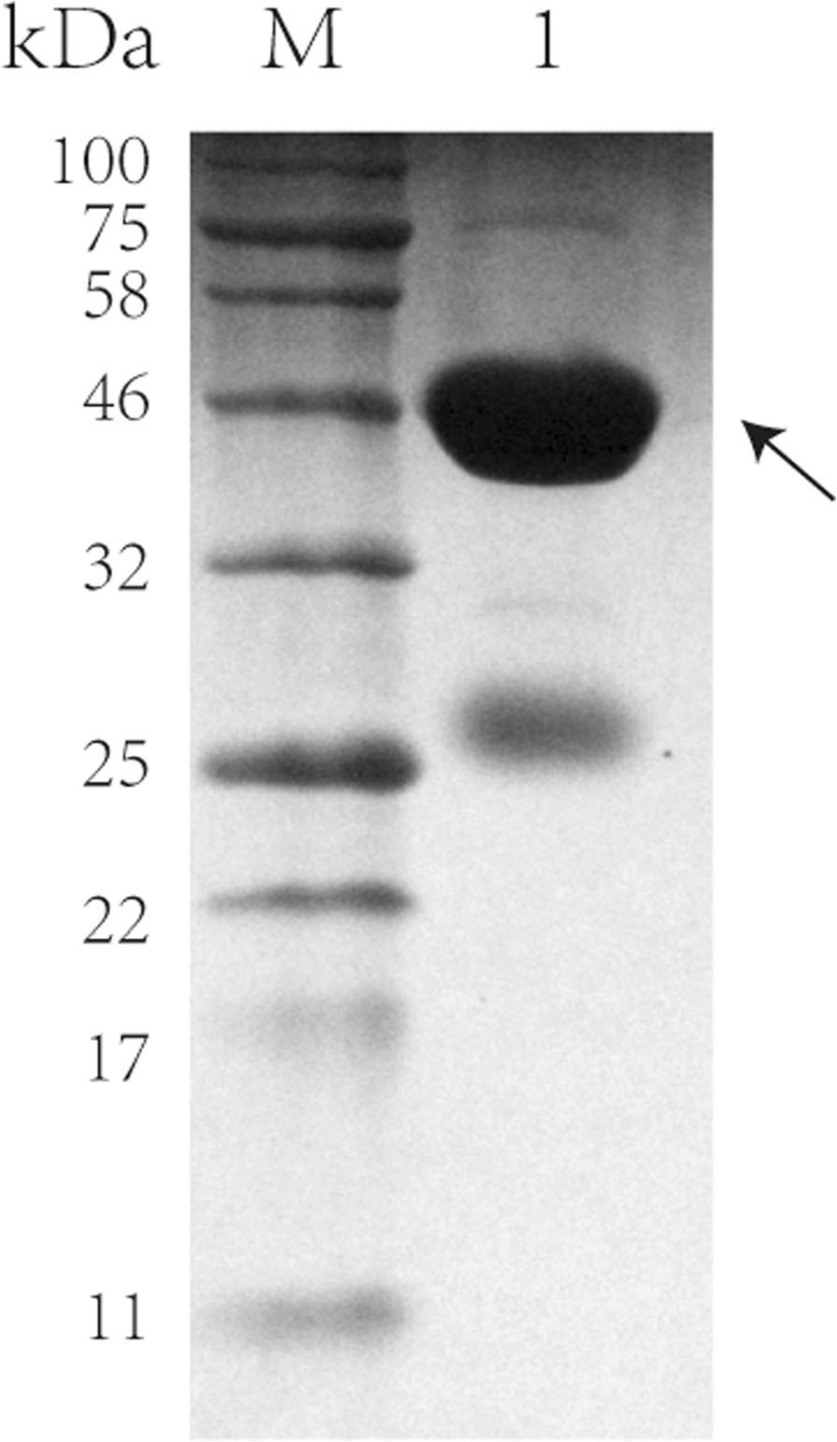
The SDS-PAGE of the GST-VP8* fusion protein. Lane M, blue prestained protein standard; lane 1, the elution of GST-VP8* protein. The arrow indicated the protein of interest. The 26 kDa protein was free GST

For the functional assay, the VP8* protein of human P [10] was expressed as a GST fusion protein (GST-VP8*). The GST-VP8* fusion protein was expressed in soluble form in *E.coli*, and the molecular weight was ~ 46 kDa (Fig. 1).

### Assay of P [10] VP8* protein binding to saliva samples

**Fig. 2:**
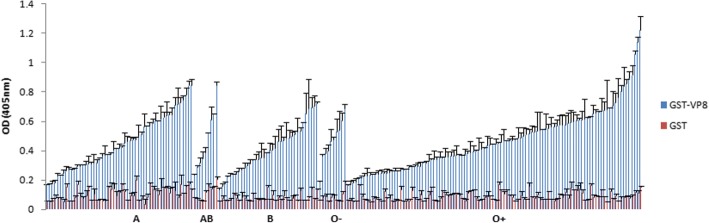
Characterization of saliva binding signals of RV P [10] VP8* based on ABO typing of saliva. Two hundred and fourteen saliva samples with known secretor A, B, O and nonscretors (O-) types were used. GST protein was included as a negative control. The cutoff value of positive samples was determined as 0.25. The blank control well was PBS instead of saliva samples. OD 450 nm, optical density at 450 nm and the error bars represent the standard deviation for each sample tested in triplicate

We first measured the binding patterns of P [10] VP8* to A-, B-, and O-type saliva. The purified GST-VP8* protein was tested in a saliva binding assay using saliva samples with known A, B, O secretors and non-secretors types. The saliva samples from 214 individuals were collected in Guangdong province including 53 individuals with A-type, 36 individuals with B-type, 9 individuals with AB-type, 106 individuals with O secretors type, and 10 individuals with non-secretors type. Human P [10] VP8* protein showed binding to saliva of all A, B, AB, O secretors and non-secretors types and GST protein did not reveal binding activity as negative control (Fig. 2).

### P [10] VP8* recognizes mucin cores

**Fig. 3:**
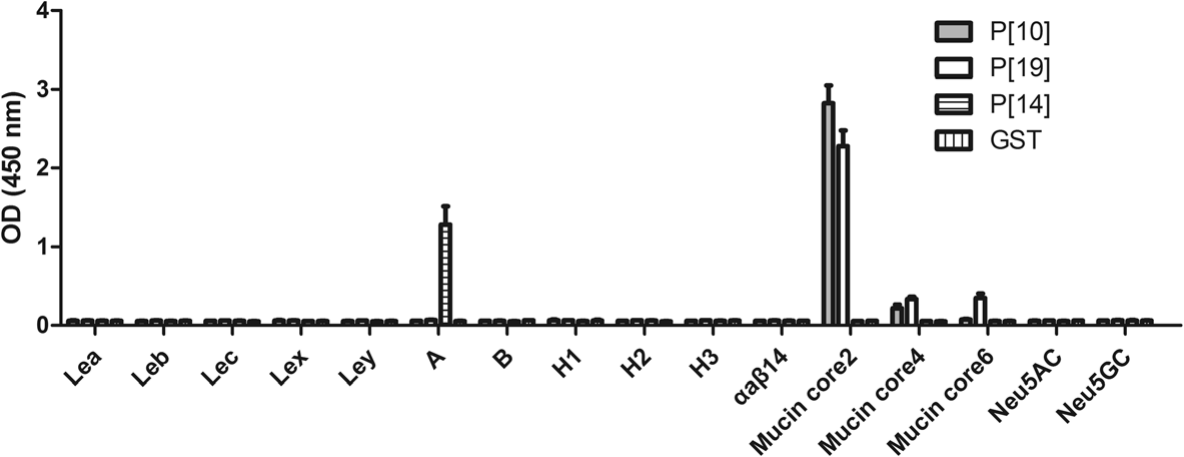
Oligosaccharide binding assay of human RV P [10] VP8* protein and P [19] VP8* protein. P [14] VP8* protein that binds to A-HBGA was used as a positive control. GST protein was used as a negative control. The error bars indicate standard deviations

In order to identify the specific binding ligand of P [10] VP8*, we conducted the synthetic-oligosaccaride binding assay. The results showed that the human P [10] VP8* protein bound strongly to mucin core 2 and weakly to mucin core 4. VP8* protein displayed no detectable binding to other syntetic oligosaccarides, such as Le^a^, Le^b^, Le^c^, Le^x^, Le^y^, A, B, H1, H2, H3, α_a_β_1,4_, mucin core 6, Neu5Ac, Neu5Gc (Fig. 3). Liu et al. has reported that P [10] and P [19] VP8*s shared nearly identical glycan binding specificity [23]. In our study, P [19] VP8* protein bound to mucin core 2 and weakly to mucin core 4 and mucin core 6, which was similar with that of P [10] VP8* protein. Human P [14] RVA has been confirmed to recognize type A HBGAs [16]. As a positive control, P [14] VP8* protein also bound to type A HBGA in our study. GST protein was used as a negative control.

### Molecular homology modeling investigation of P [10] VP8*

**Fig. 4:**
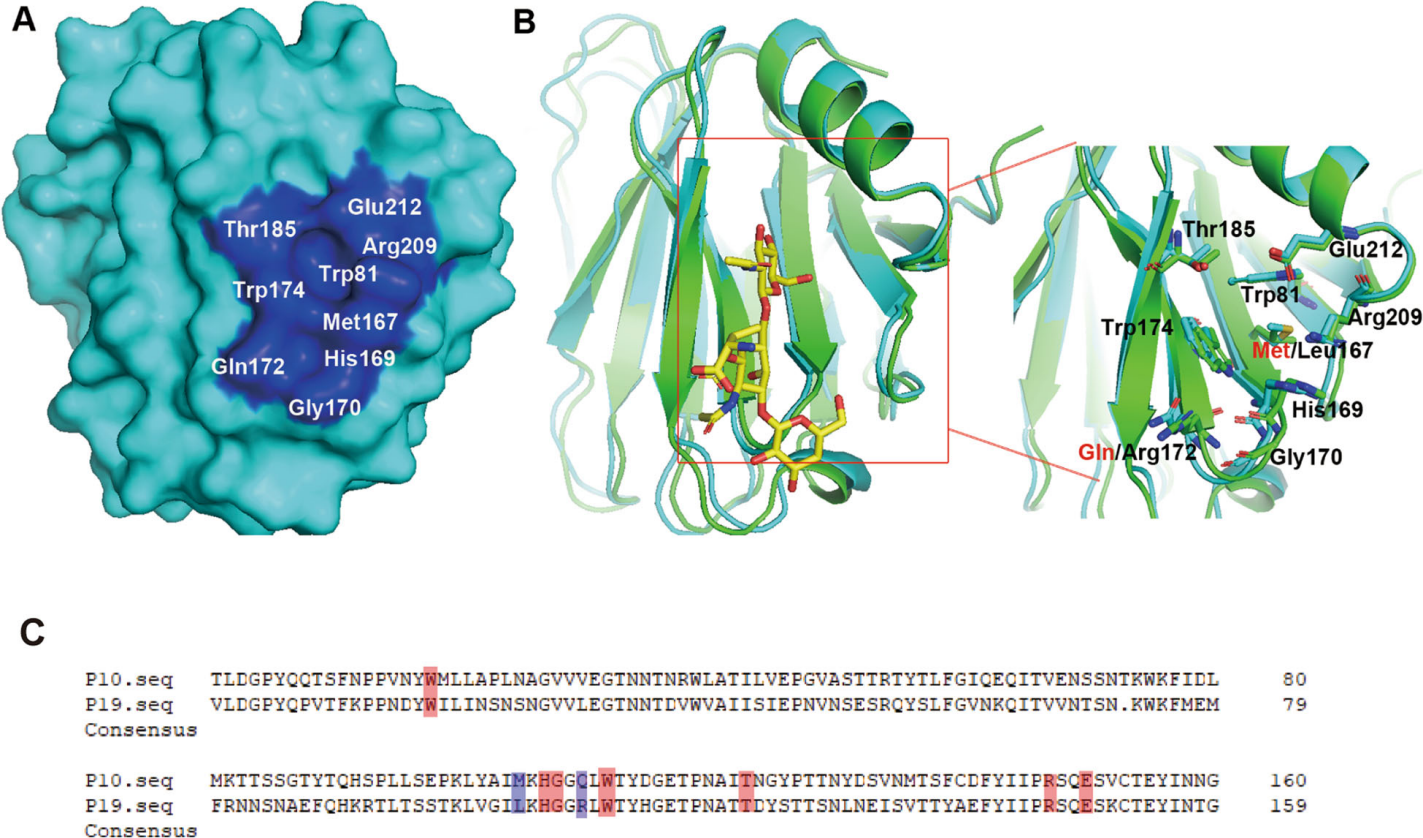
Homology modeling of P [10] VP8* and structural analysis. **a** The homology model for P [10] VP8* was shown in surface representation (colored cyan). The surface amino acid residues corresponding to the glycan binding site in P [19] VP8* are underlined and colored blue. **b** Superimposition of P [10] VP8* (cyan) with P [19] VP8*-core 2 (green; PDB ID 5VKI). The core 2 binding interfaces for the two genotypes and the amino acids involved are shown. The two residues (Met167, Gln172) in P [10] VP8* that was different to those in P [19] VP8* (Leu167,Arg172) were colored red. **c** Sequence alignment of VP8* proteins of RV P [10] and P [19]

To explore the possible docking structural basis of P [10] VP8* and mucin cores, we constructed the homology model for P [10] VP8* core protein based on the X-raycrystal structure of the Rhesus Rotavirus VP4 Sialic Acid Binding Domain in Complex with 2-O-methyl-alpha-D-N-acetyl neuraminic acid (SMT ID: 1kqr.1) as the template and the sequence identity is 74.21% (Fig. 4a). Recently, the analysis of glycan specificity of P [19] rotavirus suggested that P [10] might have a similar glycan binding pattern to that of P [19] VP8* [23, 24]. The homology model for P [10] VP8* presented a similar glycan binding cavity to P [19] VP8*, consisting of amino acids Trp81, Met167, His169, Gly170, Gln172, Trp174, Thr185, Arg209, and Glu212. Superimposition comparison of the mucin core 2 binding site in P [10] and P [19] indicated that the amino acids involved showed almost the same conformation, though two residues, Met167 and Gln172, are different in P [10] VP8* (Fig. 4b and c). Moreover, according to the previous report, the main interaction between VP8* and core 2 focused on residues Gly170, Thr185, Arg209, and Glu212, which formed hydrogen bonds with core 2 and are the same in P [10]/P [19] VP8*s.

## Discussion

Group A rotaviruses are a major cause of acute childhood diarrhea. The human P [10] rotavirus strains were in association with strains of various genotypes. So far, few isolates of rotavirus P [10] have been reported in the literature. The molecular evolution of rotavirus P [10] remains unclear. The characterization of recognition patterns of P [10] with cellular glycans is required to elucidate the origin of RV P [10] and understanding on the epidemiology of the virus. In this study, We performed different assays to explore the receptor binding specificity of P [10] RV VP8*, including the oligosaccharide binding assay, saliva-based binding assay, and structural modeling.

Significant advancements have been made in elucidating RV and host interactions. Recent reports discovered that RVs recognize HBGAs as attachment factors or receptors. HBGAs are carbohydrates that distribute abundantly on mucosal epithelia of the body, and as soluble oligosaccharides in most body fluids except cerebrospinal fluid. HBGAs are polymorphic with different ABO, Lewis and secretor versus non-secretor types. RV-HBGA interactions correlate with the host susceptibility of RV infection and illness. RVs P [8] and P [4] preferably infect individuals who were secretors/partial secretors [15]. Distinct genotypes of RVs recognize different HBGAs [25–27]. In this study, P [10] RV VP8* bound to A-, B-, AB-type, secretor or non-secretor saliva samples, implying that P [10] RVs may have a broad binding range which was similar to the previous report [23]. In the study by Liu et al., P [10] VP8* bound a low proportion of samples with no correlation to the ABH or Lewis types of the saliva donors. In our study the proportion of samples that bound with P [10] VP8* protein was high compared with previous study. We used the full sequence of P [10] VP8* for expression of VP8* protein, which was different with previous report. In addition, the VP8* sequences may be derived from different human RV P [10] strains and there maybe exist the potential difference. Otherwise, there exists other unknown attachment factors that still need to be explored to fully characterize the receptor binding specificity of P [10] RV.

Mucins are the main structural components of mucus. Mucin core 2 and mucin core 4 are commonly found in intestinal mucins. In humans, gastric and duodenal mucins generally contain the core 1 (Galβ1–3GalNAcα1-Ser/Thr) and the core 2 (Galβ1,3 (GlcNAcβ1,6) GalNAcα1-Ser/Thr) structures, and the core 3 (GlcNAcβ1,3GalNAcαSer/Thr) and core 4 (GlcNAcβ1,6 (GlcNAcβ1,3) GalNAcαSer/Thr) structures are predominant in the colon [28]. Mucin glycans are reported to be important in the cell attachment of P[II] RVs [19, 24]. Our result showed that P [10] VP8* protein recognized mucin core 2 and slightly mucin core 4 which was similar with that of P [19] VP8* protein. These data demonstrated that mucin glycans may be related with rotavirus P [10] infection.

The binding characterization of RV and cellular receptors plays a role in the cross-species transmission of RVs. The P [10] genotype belongs to the P[I] genogroup of group A rotaviruses. Most of RV genotypes in genogroup P[I] mainly infect animals, while the rotavirus P [10] strains were mainly identified from human infection [6, 8–10, 29]. Previous studies have reported that human RV P [19] belonging to P[II] genogroup interacted with mucin core 2 [19, 24]. P [10] RV VP8* binds to mucin core 2 using a potential glycan binding site that may be the same to P [19] VP8*. The homology modeling indicated that though two residues are different, all the residues displayed similar conformation and the four residues involved in hydrogen bonding interactions are the same, implying that P [10] VP8* may possess the same binding site and binds to mucin core 2 using similar mechanism. Sequence alignment showed that all P [10] strains had identical amino acid compositions on the deduced ligand binding site (data not shown). According to previous reports, alignment of the group A RV VP8* sequences showed that the amino acids of the mucin core 2 binding interface of the P [19] RVs are conserved among some other genotype RVs (P [4], P [6], P [8], P [10], and P [12]) [24]. In the previous report by Liu et al., the identical glycan binding profiles was indicated for P [10] and P [19] RVs in the glycan array. Our study confirmed the suggestion by oligosaccharide binding assay and the homology modeling. Taken together, these data indicates that mucin core 2 may play an important role in the RV epidemiology and evolution.

## Conclusions

Human rotavirus P [10] VP8* protein bound to all ABO secretor and non-secretor saliva and could bind strongly to mucin core 2 and weakly to mucin core 4. P [10] VP8* protein binds to mucin core 2 using same potential glycan binding site with that of P [19] VP8* protein.

## Abbreviations

GST: Glutathione S-transferase
HBGAs: Histo-blood group antigens
HRP: Horseradish peroxidase
IPTG: Isopropyl-β-D-thiogalactopyranoside
NSP: Nonstructural protein
PBS: Phosphate-buffered saline
RV: Rotaviruses
SDS-PAGE: Sodium dodecyl sulfate-polyacrylamide gel electrophoresis
TMB: Tetramethylbenzidine
